# An analysis of variants in *TARDBP* in the Korean population with amyotrophic lateral sclerosis: comparison with previous data

**DOI:** 10.1038/s41598-023-45593-3

**Published:** 2023-11-01

**Authors:** Wonjae Sung, Jin-Ah Kim, Yong Sung Kim, Jinseok Park, Ki-Wook Oh, Jung-Joon Sung, Chang-Seok Ki, Young-Eun Kim, Seung Hyun Kim

**Affiliations:** 1 Department of Neurology, College of Medicine, Hanyang University, Wangsimni-ro 222, Seongdong-gu, Seoul, 04763 Republic of Korea; 2 Department of Translational Medicine, College of Medicine, Seoul National University, Seoul, Republic of Korea; 3 GC Genome, Yongin, Republic of Korea; 4 Department of Laboratory Medicine, College of Medicine, Hanyang University, Wangsimni-ro 222, Seongdong-gu, Seoul, 04763 Republic of Korea

**Keywords:** Genetics, Neurology

## Abstract

The *TARDBP* gene variant is a known major cause of amyotrophic lateral sclerosis (ALS), with limited reports of Korean patients with ALS harboring the variants in *TARDBP*. This large cohort study introduces four ALS patients who share the p.M337V variant of the *TARDBP*, allowing for an investigation of clinical characteristics and prognosis by analyzing previously reported cases with the same variant. From November 2014 to August 2022, participants were recruited from two tertiary hospitals in Seoul, Korea. Clinical characteristics of patients diagnosed with ALS carrying the variant in *TARDBP* were evaluated. Previous articles demonstrating subjects’ characteristics were reviewed. Four patients were identified with the pathogenic missense variant (c.1009A>G; p.M337V) in the *TARDBP*. The mean age of onset was 55 years old, and none of the patients showed severe cognitive impairment. Sixty-three patients carrying the p.M337V variant in *TARDBP* from this study and previous reports delineated young age of onset (51.6 years), high frequency of bulbar onset patients (61.9%), and low comorbidity of frontotemporal dementia. This study reveals the presence of pathogenic variant of *TARDBP* in Korea and emphasizes the importance of genetic screening of the *TARDBP* gene, in diagnosing ALS and evaluating prognosis among familial and simplex ALS patients in Korea.

## Introduction

Amyotrophic lateral sclerosis (ALS) is a fatal, progressive neurodegenerative disease with a survival of about 3–5 years after symptom onset^1^. The clinical course of ALS is typically characterized by spreading muscle weakness and atrophy due to motor neuron degeneration in the brain and spinal cord^2^. However, up to 60% of patients exhibit cognitive and behavioral changes, and a quarter of them are diagnosed with frontotemporal dementia (FTD)^3^. The discovery of common pathological phenomena of ALS and FTD, such as transactive response DNA binding protein with a molecular weight of 43 kDa (TDP-43) proteinopathy, and the improvement of genetic analysis technology extended the knowledge of complex genetics in familial and simplex ALS^4,5^. Among the more than 40 genes associated with ALS, repeat expansions of chromosome 9 open reading frame 72 (*C9orf72*), and variants in superoxide dismutase 1 (*SOD1*), fused in sarcoma (*FUS*), and TAR DNA-binding protein 43 (*TARDBP*), are the most frequent genes identified in ALS^4,6^.

The *TARDBP* gene is located on chromosome 1p36 and encodes TDP-43, a highly conserved RNA/DNA-binding nuclear protein stabilizing mRNA and regulating transcription and alternative splicing^7,8^. Variant in *TARDBP* mediates TDP-43 fragmentation, which loses function and aggregates in the cytoplasm and/or nucleus of central nervous system neurons^9,10^. Pathogenic (P.V.) or likely pathogenic variants (LPV) are predominantly located in the exon six that encodes intrinsically disordered regions nearby the C-terminal of TDP-43^11^. The frequency of variants in *TARDBP* is 3.3% in familial ALS and 0.5% in simplex ALS^6^. However, only one ALS case has been reported harboring P.V. of *TARDBP*, which has been scarcely identified in Korea^12^. Therefore, the prevalence and clinical traits of Korean patients diagnosed with ALS carrying variants in *TARDBP* were unobtainable due to the rarity of the patients.

The present study conducted genetic screening for patients diagnosed with ALS in two large cohorts and addressed the clinical characteristics of patients with ALS harboring variants in *TARDBP*. Furthermore, we reviewed their distinct features of the genotype–phenotype correlation demonstrated in previous studies.

## Results

### Variant analysis of the ALS patient cohorts

Among 1130 subjects diagnosed with ALS, four patients with variants in *TARDBP* were identified in these two cohorts (4/1130, 0.35%). All individuals harboring variants in *TARDBP* carried the same variant (c.1009A>G, p.M337V), a missense variant located in the exon 6 of *TARDPB* encoding the low-complexity domain. The variant was categorized as a pathogenic variant according to ACMG and AMP guidelines.

### Clinical features of the patients with ALS carrying *TARDBP* c.1009A>G, p.M337V variant

**Figure 1:**
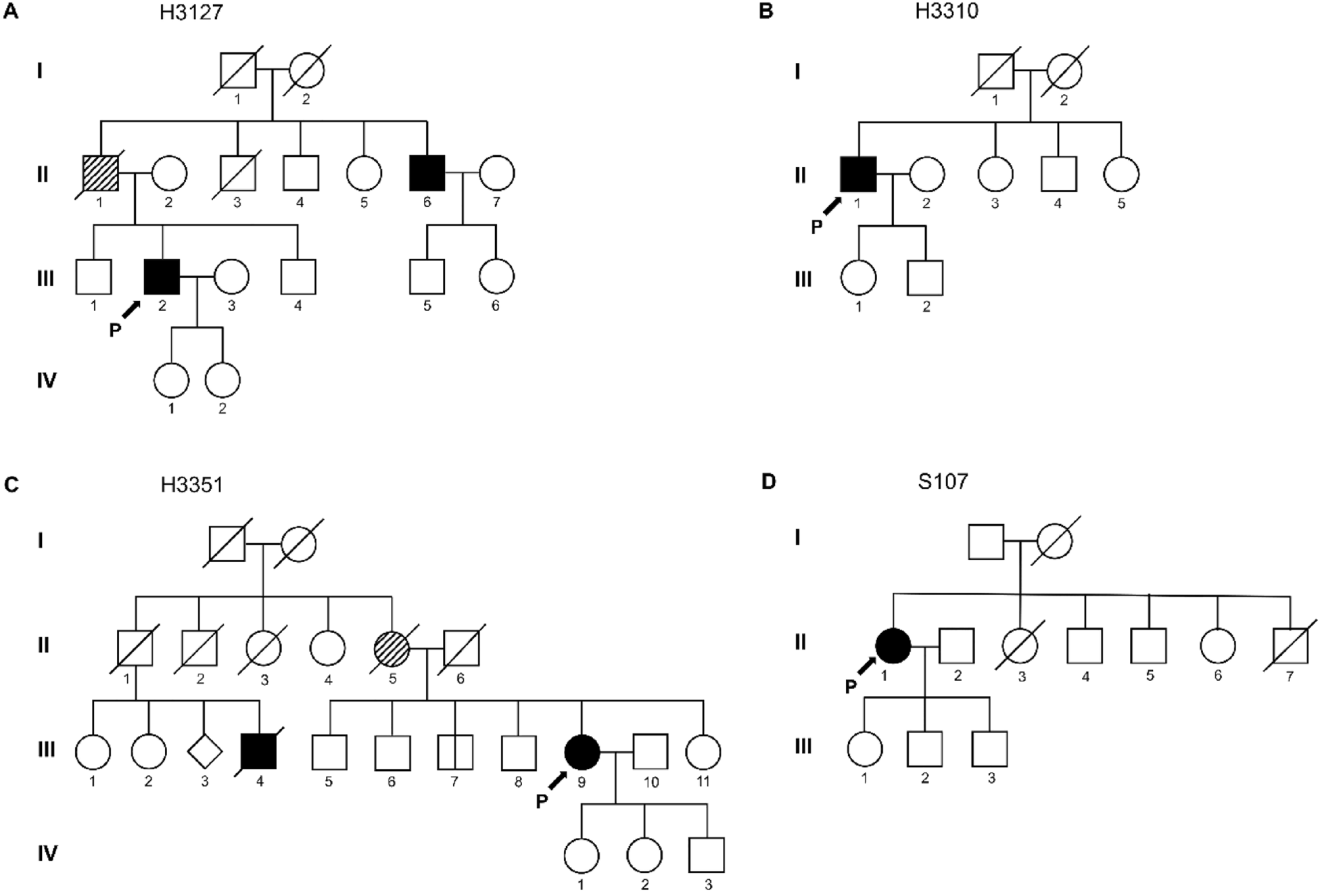
Family pedigree of patients with ALS carrying p.M337V variant of *TARDBP*. Squares represent male individuals, while circles represent female individuals. Each symbol represents a distinctive individual, identified by a specific numeric code. Roman numerals indicate the generation number (I, II, III). Individuals diagnosed with ALS are shown in solid black. The diagonal hatching symbols represent those with suspected ALS. (**A**) The proband's (III-2) father (II-1) exhibited dysarthria which can be suspected as a symptom of ALS. The proband's uncle (II-6) was diagnosed with bulbar-onset ALS without confirmation of genetic variants. (**B**) The patient's (II-1) family history denied the presence of other ALS patients. (**C**) While the family members presented a history of ALS, the patient's (III-9) brother did not present any symptoms related to ALS. (**D**) According to the patient's family history (II-1), there were no known ALS cases in the patient's family. P, Proband.

Table 1 summarizes the clinical features of four patients carrying variants in *TARDBP*. The 51-year-old male patient (H3127; Fig. 1A, III-2) initially presented with worsening dysarthria and dysphagia, followed by limb weakness. Although his father (Fig. 1A, II-1) was not diagnosed with ALS, he revealed dysarthria and atrophy in limbs. In addition, the younger brother of the patient's father (Fig. 1A, II-6) was also diagnosed with ALS manifesting the initial symptom of dysarthria at age 60. The neurological evaluation presented modest muscle atrophy and fasciculation in his tongue and limbs with enhanced tendon reflexes bilaterally. Furthermore, pseudobulbar palsy and Hoffman signs were also observed. Electromyography (EMG) demonstrated the presence of widespread denervation potentials and evidence of regeneration in muscles of the upper and lower limbs. The patient's ALSFRS-R score was 44 at the initial evaluation, and the progression rate was relatively slow (0.28). Furthermore, any cognitive impairment was not detected in neuropsychological battery tests.

**Table 1:** Clinical features of patients with ALS harboring variants in *TARDBP*.

MND	Family history	Sex/age of onset (y)	Site of onset	ALS duration (m)	Initial ALSFRS-R score	Progression rate	Cognitive status
H3127	Yes	M/49	Bulbar	35	42	0.28	Normal
H3310	No	M/61	Spinal (lower limb)	14	24	3.42	Decreased language retrieval and confrontational naming
H3351	Yes	F/49	Bulbar	17	47	0.08	Normal
S107	No	F/61	Spinal (lower limb)	90	28	0.22	Normal

ALS, amyotrophic lateral sclerosis; ALSFRS-R, amyotrophic lateral sclerosis functional rating scale-revised.

The second patient (H3310; Fig. 1B, II-1) was a 64-year-old male with progressive weakness beginning in his lower limbs over the last 3 years. He denied any family history of ALS. On neurologic examination, brisk reflexes on the left lower limb and fasciculations on whole limbs were noted. EMG results presented widespread neurogenic lesions. Consequently, he was diagnosed with clinically probable, lab-supported ALS. His symptoms worsened rapidly (progression rate = 3.42), eventually becoming bedridden. However, personality change was not observed, and cognitive impairment was minimal, demonstrating mildly decreased language retrieval and confrontational naming.

The third proband of family 3 (H3351; Fig. 1C, III-9) was a female patient diagnosed with clinically probable ALS at 50 years of age, presenting aggravations of dysarthria for a year. Her mother (Fig. 1C, II-5) also showed dysarthric speech and passed away at 53. While her mother's siblings denied diagnosis of ALS, one cousin (Fig. 1C, III-4) was diagnosed with ALS and died in 4th decade. The neurological examination showed moderate dysarthria, an atrophied tongue with fasciculation, and positive Hoffmann signs. In addition, neurogenic lesions were observed in the muscles innervated by the hypoglossal nerve, cervical, and thoracic spinal cord on EMG. Although the patient was diagnosed with familial ALS carrying the variant in *TARDBP*, her symptom exhibited very slow progression without any cognitive deficit (progression rate = 0.08).

The fourth patient (S107; Fig. 1D, II-1) was a 69-year-old woman who presented with bilateral lower extremity weakness that started in her early 60 s and gradually progressed to upper extremity weakness and dysarthria. She denied any family history of ALS. The neurological examination revealed slurred speech, brisk reflexes on bilateral upper limbs, and fasciculation on both upper and lower limbs. She had pseudobulbar palsy, but her cognitive function was normal. Widespread neurological lesions were observed on the EMG test. Finally, she was diagnosed with clinically probable ALS, 4 years after the initial onset of symptoms. She has had a very slow progression, with a disease duration of 7.5 years and a progression rate of 0.22.

While the confirmation of kinship among the subjects was not possible through pedigree verification, analysis using the Peddy software revealed relatedness estimates ranging from 0.02 (H3127-H3310) to 0.04 (H3127-S107, H3127-H3351, and H3310-H3351) for each individual, consistent with second to third cousin relationships (Supplementary Table 1).

## Discussion

In two Korean ALS cohorts, we investigated the presence of variants in *TARDBP*, and the single pathogenic p.M337V variant is exclusively confirmed in two familial and two simplex ALS cases. Two patients diagnosed with familial ALS presenting dominant bulbar weakness exhibited slow progression. In addition, cognitive impairments implicating FTD were absent in all patients carrying variants in *TARDBP*. This research demonstrated that a single type of potentially pathogenic variant in *TARDBP* exists among Koreans and described the features shared by patients carrying this variant.

The frequency of variants of *TARDBP* in the European population was 4.2% and 0.8% each in familial and simplex ALS^6^. While it has been identified as 1.5% and 0.2% each in familial and simplex ALS in Asian populations, indicating a lower frequency than in Europeans^6^. In addition, comprehensive genetic studies investigating Korean patients diagnosed with ALS showed the absence of pathogenic variants in *TARDBP*^13,14^. Only one case has described a single ALS patient carrying the p.M337V in the *TARDBP* gene, which is the same variant confirmed in the four unrelated patients in this study^12^. The rarity and lone type of variant in the *TARDBP* gene could be the distinctive characteristics in Korean patients diagnosed with ALS. While this study could not confirm the presence of a founder effect, the in-silico analysis revealed that the subjects exhibited a second to third-cousin relationship.

The missense variant c.1009 A>G (p.M337V) in *TARDBP* has repeatedly been identified in Western countries and Asia (Table 2). A recurrent p.M337V variant was detected in five family members of the British-descent Caucasian family^9^. Two patients were bulbar-onset ALS, and the other three were limb-onset ALS. These subjects' mean age of onset was 47 years (range 44–52 years), and the average disease duration was 5.5 years (range 4–7 years). These patients did not present a history of dementia. Another Caucasian carrier diagnosed with spinal-onset ALS from the U.S. also demonstrated early symptom onset (38 years) without evidence of cognitive impairment or atypical features^15^. An Italian patient with a family history of ALS was also carrying the p.M337V variant in *TARDBP*^16^. She had been diagnosed with ALS when she was 53 years old after mild motor impairment in her limbs. She survived until she was 56 years old without cognitive decline. In northern England, four Caucasian patients in one family were diagnosed with familial ALS harboring the p.M337V variant in *TARDBP*^17^. The mean age of onset was 46.5 years (range 31–57 years). Two patients developed dysarthria as the initial symptom. Affected members of this family demonstrated variable disease duration, with one member dying only 9 months after the first symptoms and the other patient dying 17 years after disease onset.

**Table 2:** Summary of demographic and clinical data of patients with ALS carrying variants in *TARDBP* in previous articles.

Reference	Ethnicity (country)	Number of patients	Age of onset, y (range)	Site of onset	Disease duration, y (range)	Cognitive status
Sreedharan et al.^9^	Caucasian (UK)	5	47 (44–52)	Bulbar: 2 Spinal: 3	5.5 (4–7)	Normal
Rutherford et al.^15^	Caucasian (US)	1	38	Spinal	NA	Normal
Corrado et al.^16^	Caucasian (Italy)	1	53	Spinal	3	Normal
Kirby et al.^17^	Caucasian (UK)	4	46.5 (32–57)	Bulbar: 2 Spinal: 2	6.9 (0.75–17)	Normal
Tamaoka et al.^18^	Asian (Japan)	6	52.5 (44–61)	Bulbar: 6	9.5 (9–10)	Normal
Tsai et al.^19^	Asian (Taiwan)	2	52 (47–57)	Spinal: 2	More than 3y	Normal
Ju et al.^20^	Asian (China)	27	51.0 (38–67)	Bulbar: 20 Spinal: 7	10.2 (5.1–18)	Cognitive impairment: 2 Normal: 17 NA: 8
Pang et al.^21^	Asian (Hong Kong)	1	52	Bulbar	13.7	NA
Xu et al.^22^	Asian (China)	10	58.4 (50–62)	Bulbar: 6 Spinal: 4	7.6 (3–14)	Normal
Narain et al.^23^	Asian (India)	1	40	Spinal	NA	Normal
Han et al.^12^	Asian (Korea)	1	50	Spinal	NA	Normal
This study	Asian (Korea)	4	55 (49–61)	Bulbar: 2 Spinal: 2	3.5 (1.0–7.5)	Normal: 3
Total		63	51.6	B:S = 39:24		

NA, not available; UK, United Kingdom; US, United States of America.

In addition, studies from Asia presented patients with ALS carrying the p.M337V variant of *TARDBP*. Six subjects in a Japanese family shared the p.M337V variant in *TARDBP*, which was segregated with the disease^18^. All patients presented dysarthria as the initial symptom with a long disease duration (9–10 years). Although some patients had a delayed onset, the mean onset age of Japanese carriers was 52.5 years (range 44–61 years). The family had neither a history of dementia nor any atypical characteristics. A study from Taiwan also demonstrated two unrelated patients with familial ALS carrying the p.M337V variant of *TARDBP*^19^. Patients exhibited limb weakness as the first symptom at 47 and 57, also showing an early age of onset. One patient revealed a long survival duration which was over 60 months. Furthermore, various studies from China reported many patients with the same variant in *TARDBP*, including a large family with multiple patients diagnosed with ALS^20–22^. The family included 18 patients confirmed to harbor the p.M337V variant in *TARDBP* and nine patients diagnosed with ALS without genetic evaluation. Their mean age of onset was 51.0 (range 38–67 years), and the mean survival period was about 10 years (range 61–216 months). While most patients denied any history of dementia, two presented cognitive impairment^20^. Two additional Asian subjects in Hong Kong and India exhibited relatively young age-onset ALS^21,23^.

When investigating the clinical characteristics of all patients with the p.M337V variant of *TARDBP* in the East and West, the patients were diagnosed at a relatively young age, and the incidence of bulbar-onset ALS was higher than the previously reported incidence (mean age of onset: 51.6, Bulbar: spinal ratio = 39:24)^24^. Furthermore, the disease duration was longer than the median survival period of general patients diagnosed with ALS, which ranges from 2 to 4 years^25^. These findings were consistent with the previous studies, which analyzed genotype–phenotype correlations in Asian patients with variants in *TARDBP*^26,27^. Although the onset age was older than that of patients carrying the p.M337V variant of *TARDBP* in previous articles, subjects reported in this study also presented younger age of onset than the general Korean ALS patients (56.5 vs. 61.4)^28^. In addition, two patients with bulbar-onset ALS demonstrated a slow progression, possibly resulting in prolonged disease duration. The absence of FTD was also consistent with previous studies reporting a low prevalence of FTD. Although data on a limited number of individuals were available, it was determined that they exhibited symptoms like those of ALS patients with the p.M337V variant in *TARDBP*.

The present study had several limitations. First, the pathologic presentation of the variant is absent. Autopsies to investigate pathological changes in anterior horn cells were impossible since no deceased patients were available for study. Nonetheless, a prior study demonstrated that experiments utilizing a mouse model with the p.M337V variant in *Tardbp* revealed motor neuron degeneration attributed to partial aberrant splicing alterations and compromised stress granule dynamics without the cytoplasmic TDP-43 aggregation^29^. Although direct pathological confirmation is lacking, given that pathognomonic mechanisms have already been elucidated and the variant is recognized as pathogenic, the genetic variant has likely contributed to the disease. Second, the estimation of age-dependent penetrance was unavailable. This limitation arises from the absence of data on asymptomatic carriers with the p.M337V variant in *TARDBP*. The presymptomatic testing for evaluating variants in the *TARDBP* gene has not been conducted in Korea, including this study. Previous literature based on other populations also lacked information on asymptomatic carriers. Further research should focus on predicting age-dependent penetrance by verifying individuals within pedigrees with a definite family history who carry pathogenic variants of *TARDBP* but remain asymptomatic. Lastly, determining the presence of founder effect was impossible. Since subjects only underwent WES, it was impossible to conduct additional studies for haplotype analysis. Only a single pathogenic variant in the *TARDBP* has been identified in this study. Furthermore, analysis based on WES data using relevant software packages has revealed that the subjects exhibit second to third-cousin relationships. Consequently, further research is essential to investigate the presence of a founder effect.

Despite these limitations, we identified a significant number of ALS patients harboring the p.M337V variant of *TARDBP* in the Korean population by performing screening in two cohorts. Our results support the different genotypic spectrum of ALS between Europeans and East Asians. Furthermore, this study enriches our understanding of clinical characteristics sharing the p.M337V variant in the *TARDBP* gene, which tend to early disease onset, delayed disease progression and a low prevalence of FTD.

## Material and methods

### Patients

From November 2014 to August 2022, all participants in this study were recruited from the ALS clinic of Hanyang University Hospital and Seoul National University Hospital in Seoul, Korea. According to the El Escorial Revised Criteria, all patients met the diagnostic criteria for possible, probable, laboratory-supported, or definite ALS^30^. Patients with diagnoses of pure upper motor neuron or pure lower motor neuron phenotype were excluded for establishing a more refined disease category for ALS. The clinical characteristics such as sex, family history of ALS, age of onset, site of onset, disease progression, electromyography (EMG) findings, and motor function score measured as ALSFRS-R (0–48) were analyzed. The progression rate from symptom onset to the time point of the initial evaluation was calculated as follows:

$$\mathrm{Progression \; rate }=\frac{48 - (\mathrm{ALSFRS-}\mathrm{R \; at \; initial \; evaluation})}{\mathrm{the\; time \; interval\; from \;symptom \;onset \;to \;the \;first \;evaluation }(\mathrm{months})}$$

Three subjects (H3127, H3310, H3351) underwent the Seoul Neuropsychological Screening Battery (SNSB), a widely employed neuropsychological assessment tool in Korea, to evaluate their behavioral and cognitive status. When the SNSB assessment was not conducted (S107), the Korean version of the Mini-Mental State Examination and the Frontal Assessment Battery were applied^31^.

This study was approved by the Institutional Review Board of Hanyang University Seoul Hospital and Seoul National University Hospital (HY2013-11–025 and IRB 1904–165-1031, respectively). Every patient and/or their legal caregivers provided written informed consent before being included in the research. This study was carried out in accordance with the Declaration of Helsinki.

### Genetic analysis

Genomic DNA was extracted from peripheral blood leukocytes using a Wizard Genomic DNA purification kit according to the manufacturer's instructions (Promega, Madison, WI). Whole exome sequencing was performed using Agilent SureSelect all Exon kit 50 Mb (Agilent, Santa Clara, CA). Sequencing libraries were prepared according to the manufacturer's instructions, and the flow cell was loaded on a NextSeq 500 sequencing system (Illumina) for sequencing with 2 × 100 bp read lengths. Reads were mapped to the GRCh37/hg19 build using the Burrows-Wheeler Aligner (BWA), and variants were called using GATK software. In-silico analysis was applied using dbNSFP^32^. We filtered out all variants with allele frequencies > 0.001 based on various public databases, including the gnome Aggregation Database (gnomAD, http://gnomad.broadinstitute.org/) and the Korean Reference Genome Database (http://coda.nih.go.kr/coda/KRGDB/index.jsp). We screened exons and flaking regions of 75 ALS-FTD related genes (Supplementary Table 2).

We utilized the Peddy software package (https://github.com/brentp/peddy), which can identify and correct errors in DNA sequencing investigations^33^. Relatedness was inferred by analyzing individual genotypes derived from whole-genome or whole-exome sequencing.

### Literature review

We searched the literature to review previous articles discussing patients with ALS carrying the p.M337V variant in *TARDBP*. We searched PubMed.gov on March 2023 for "TARDBP" or "TDP-43" and "mutations" or "variants" and with the MeSH terms "amyotrophic lateral sclerosis" or "ALS." To exclude experimental studies, we excluded "pathology" while specifying subjects as "humans." The search was limited to publications dating from 2008 to 2020. The literature search identified 310 articles. Of these, 13 articles reported on a series of patients harboring the p.M337V variant in *TARDBP*. Eleven articles were reviewed after excluding two research without descriptions of patients' clinical features. We reviewed the clinical characteristics of the patients carrying the p.M337 variant in *TARDBP*, mainly focusing on ethnicity, onset age, site of onset, disease duration, and cognition.

### Supplementary Information

Supplementary Tables.

## Data Availability

The authors confirm that the data supporting the findings of this study are available within the article and its supplementary material. The datasets generated and/or analysed during the current study are available in the ClinVar repository, SCV004023203.
