# Supplementary material for: An analysis of variants in TARDBP in the Korean population with amyotrophic lateral sclerosis: comparison with previous data
Source: Sci Rep. 2023 Nov 1;13:18805. doi: 10.1038/s41598-023-45593-3 (PMC10620191; doi:10.1038/s41598-023-45593-3)
Supplement: Supplementary file 1 — Supplementary Tables. [file 41598_2023_45593_MOESM1_ESM.pdf]

An Analysis of Variants in *TARDBP* the Korean Population with Amyotrophic Lateral Sclerosis: comparison with previous data

Wonjae Sung[1,†], Jin-Ah Kim[2,†], Yongsung Kim[1], Jinseok Park[1], Ki-Wook Oh[1], Jung-Joon Sung[2], Chang-Seok Ki[3], Young-Eun Kim[4,*], and Seung Hyun Kim[1,**]

Authors' Institutions :

1 Department of Neurology, College of Medicine, Hanyang University, Seoul, Republic of Korea

2 Department of Translational Medicine, College of Medicine, Seoul National University, Seoul, Republic of Korea

3 G.C. Genome Corporation, Yongin, Republic of Korea

4 Department of Laboratory Medicine, College of Medicine, Hanyang University, Seoul, Republic of Korea

† Indicates equal contribution

* Corresponding author at: Young-Eun Kim, Department of Laboratory Medicine, College of Medicine, Hanyang University, Seoul, Republic of Korea. Tel.: +82-2290-8974.
E-mail: young0eun@hanyang.ac.kr

** Corresponding author at: Seung Hyun Kim, Department of Neurology, College of Medicine, Hanyang University, Seoul, Republic of Korea. Tel.: +82-2290-8371.
E-mail: kimsh1@hanyang.ac.kr

Supplementary table 1. Relatedness within subjects carrying the p.M337V variant in the *TARDBP*

	H3127	H3310	H3351	S107
H3127		0.02311	0.03747	0.03659
H3310			0.03552	0.0251
H3351				0.03279
S107				

Supplementary table 2. Gene list of ALS-FTD

Disease	Genes
ALS-FTD	*ALS2, ANG, ANXA11, APEX1, ARHGEF28, C21ORF2, CAMTA1, CCNF, CHCHD10, CHCHD2, CHMP2B, CHRNA4, CYLD, DAO, DCTN1, DNAJC7, DPP6, ELP3, ERBB4, EWSR1, FAM160B1, FIG4, FUS, GGNBP2, GLE1, GLT8D1, GRN, HFE, HNRNPA1, HNRNPA1P10, HNRNPA2B1, HNRNPH2, ITPR2, KIAA1217, KIAA1600, KIF5A, KIFAP3, MAPT, MATR3, MOBP, NEFH, NEK1, NT5C3B, NT5C3L, OPTN, PFN1, PLCD1, PON1, PON2, PON3, PRNP, PRPH, SCFD1, SETX, SIGMAR1, SOD1, SPG11, SPTLC1, SQSTM1, SRCAP, SS18L1, TAF15, TARDBP, TBK1, TET2, TIA1, TREM2, TRPM2, TUBA4A, UBQLN2, UNC13A, VAPB, VCP, VEGFA, ZNHIT3*

ALS, amyotrophic lateral sclerosis; FTD, frontotemporal dementia
